# Real-based Polarity-preserving Asymmetric Fourier Imaging (RepAFI)

**DOI:** 10.2463/mrms.tn.2015-0152

**Published:** 2016-07-29

**Authors:** Tokunori Kimura, Hiroshi Kusahara

**Affiliations:** 1 Clinical Application Research and Development Department, Center for Medical Research and Development, Toshiba Medical Systems Corporation, 1385 Shimoishigami, Otawara, Tochigi 324-8550, Japan; 2 MRI Systems Development Department, MRI Systems Division, Toshiba Medical Systems Corporation

**Keywords:** asymmetric Fourier imaging, partial Fourier imaging, magnetization polarity, vessel wall imaging, FLAIR

## Abstract

We proposed and assessed a modified asymmetric Fourier imaging (AFI) technique named real-based polarity-preserving AFI (RepAFI), in which the low-pass filter kernel for background phase estimation in AFI is optimized to preserve the magnetization polarity information for blood vessels and cerebrospinal fluid (CSF) even for data obtained using phase-sensitive inversion-recovery spin-echo-based (PSIR-SE) sequences with asymmetrical sampling in the k-space. Our proposed RepAFI technique achieves a practical balance of image quality and simplicity to provide better performance than conventional AFI methods.

## Introduction

Phase-sensitive and real-part imaging techniques that use an inversion-recovery (IR) sequence (PSIR/Real-IR) in combination are useful for T_1_-weighted (T_1_W) imaging,^1^ fluid-attenuated inversion-recovery (FLAIR) imaging,^2^ and black-blood (BB) imaging,^3^ because they provide images in which the magnetization polarity is preserved. The preservation of the magnetization polarity is particularly vital when the longitudinal magnetization (*M*_z_) polarities (positive and negative) are mixed as a result of the inversion time (TI), being set to a value that is smaller than the TI for nulling *M*_z_ (TI_null_). However, background phase correction is usually needed to produce correct PSIR images, even for spin-echo (SE) or fast-spin-echo (FSE) sequences that are free from the susceptibility-dependent phase artifacts that are seen in gradient-echo sequences. Therefore, other data that ensures positive *M*_z_ in whole tissue is used for phase correction in PSIR sequences. This data is obtained without the inversion pulse or with TI > TI_null_ in PSIR sequences. In addition, the symmetric k-space sampling is required to prevent the introduction of phase errors in phase correction as will be shown later in theory section.

On the other hand, a standard asymmetric Fourier imaging (AFI) or a partial Fourier imaging (PFI) reconstruction technique for asymmetrically sampled k-space data in magnetic resonance (MR) imaging can produce images that are almost equivalent to the images obtained from symmetrically sampled full k-space data based on the Hermitian conjugate theory.^4^ These techniques have been widely used to reduce imaging time or shorten TE while minimizing blurring. Several AFI algorithms have been proposed and applied.^5–8^ The simplest algorithms include the Margosian^5,6^ and homodyne^7^ techniques, which are equivalent, in which the phase is estimated using symmetric portions of the low frequency parts of the k-space self-data. The projection on to convex sets (POCS) technique^8^ and the Cuppen technique,^9^ in which iteration is performed under the assumption that the real-space (r-space) image contains only the real component after phase correction, can reduce the artifacts induced by the Margosian and homodyne techniques at the cost of computing time.^10^ Xu et al. has extended the POCS algorithm to 2-dimensional (2D) partial sampling,^11^ and several approaches have been proposed combining AFI with parallel imaging (PI) techniques that allow acceleration using multichannel coil data.^12^ In a more recently proposed magnitude-based AFI technique, the phase information is not required and the phase-dependent errors are lower than in conventional methods.^13^

However, none of the proposed AFI algorithms can be applied to data containing positive and negative signals, such as PSIR data, because MR signals are assumed to be all positive in standard AFI due to the phase estimation process. In standard AFI, the phase estimation is commonly performed using a low-pass filter kernel determined based only on the sampled data size in k-space without considering the spatial frequency components of the background phase, resulting in unstable signal polarities when the algorithm is applied to PSIR data.

In this study, we have proposed and assessed a modified AFI technique named real-based polarity-preserving AFI (RepAFI), in which the magnetization polarity can be preserved even for PSIR sequences with asymmetric k-space sampling by optimizing the low-pass filter kernel used for phase correction in standard AFI algorithms.

## Theory

**Fig 1.**
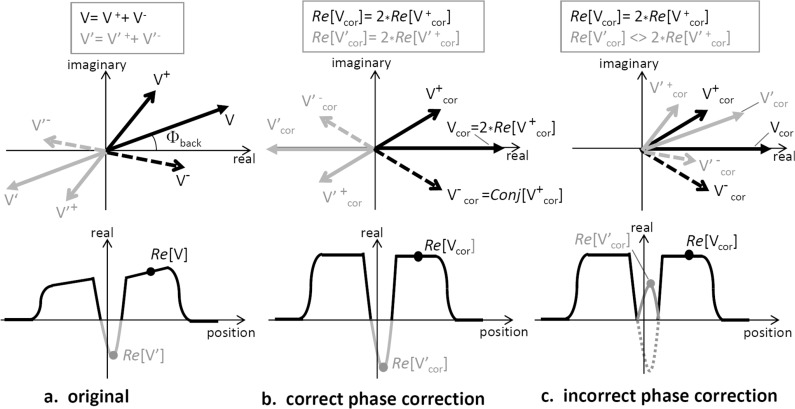
Principles of real-based polarity-preserving asymmetric Fourier imaging (RepAFI) for inversion-recovery (IR) signals with polarity. Here, it is assumed that the positive background signal is *V*, and the negative blood vessel signal is *V′*. The actual magnetic resonance (MR) signals are complex and include the additional artifactual background phase ***Φ***_back_ (**a**). In AFI, the unknown signal *V*^−^ (corresponding to data in the unsampled k-space region) must be estimated from the known signal *V*^+^ (corresponding to data in the sampled k-space region). If the pure background phase ***Φ***_back_ can be estimated and then corrected, the intensity of the ideal signal *V* (= *V*^+^ + *V*^−^), including the polarity, can be obtained by doubling the real part of *V*^+^ (**b**). However, the correct signal intensity, especially for negative polarities, cannot be obtained if the measured ***Φ***_back_ is incorrect (**c**). The purpose of RepAFI is to enable acquisition of the correct signal, including the polarity, by enabling correct measurement of ***Φ***_back_.

First, the principles of standard AFI are reviewed. As can be seen in Fig. 1, an ideal MR signal *V* is assumed to be a vector summation of the complex conjugate signals *V*^+^ and *V*^−^. In AFI, the unknown *V*^−^ (corresponding to data in the unsampled k-space region) is estimated from the known *V*^+^ (corresponding to data in the sampled k-space region). However, the actual MR signals include the additional artifactual background phase *Φ*_back_. For symmetrically sampled data for the full k-space with positive polarity, the ideal *V* is obtained simply by obtaining the magnitude even if *Φ*_back_ is unknown*.* In contrast, for asymmetrically sampled data for the full k-space (*V*^−^ unknown), if *Φ*_back_ can be estimated from the self-data and then corrected, *V* can be obtained by doubling the real part of *V*^+^, and the polarity becomes correct. However, the correct *V* cannot be obtained regardless for the full or partial sampling if the polarity is negative, and the measured *Φ*_back_ is incorrect. The purpose of RepAFI is to enable acquisition of the correct *V*, including the polarity, by enabling the correct measurement of *Φ*_back_.

Second, we consider the case where the positive and negative signals are mixed when there is no other signal whose phase is 0 or |*π*| at the ideal condition of *Φ*_back_ = 0. This can happen, for example, when the TI of the PSIR sequence is shorter or longer than the TI_null_*S* of the different tissues under the condition of mixing of several tissues with different T_1_ values. The phases of the positive and negative signals are respectively *Φ*_back_ and *Φ*_back_ + |*π*|. A basic assumption in the RepAFI technique is that the background phase *Φ*_back_ can be separated from the measured total phase while preserving the polarity information in the r-space corresponding to the sampled self-data in the k-space. To preserve the magnetization polarity by eliminating only *Φ*_back_ in the AFI algorithm, separation should be possible.

Does the use of spatial frequency differences, for example by windowing in the k-space, make separation possible? Fortunately, the background phase changes smoothly, and is concentrated in the low frequency part of the k-space, especially for SE- and FSE-based sequences (the high-frequency components in the background phase are significant for GRE-based sequences). The blood vessel and CSF sections have longer T_1_ (thus longer TI_null_) than those for the stationary tissues (gray matter, white matter, fat, muscle, etc.), and are also regarded to be relatively small spatially, concentrated in the high-frequency part of the k-space. If such conditions can be assumed, the background phase is expected to be separated in SE-based IR sequences even at TI > TI_null_ for stationary tissues and TI < TI_null_ for blood or CSF by using optimal low-pass windowing in the k-space in standard AFI algorithms. RepAFI is a modified AFI technique in which the low-pass filter for phase correction in standard AFI algorithms is optimally designed to correctly estimate the background phase even under the above conditions.

RepAFI is expected to be effective even for asymmetrically sampled data obtained with PSIR sequences. Alternatively, if *Φ*_back_ is estimated correctly using separate data (extra data) without using IR, the polarity and structure can be restored perfectly, in the same manner as for fully sampled data.

### RepAFI algorithm

Here, as similarly as our proposed another AFI technique named *MagAFI*,^13^ we provide a simplified explanation of Rep-AFI using the case of 1D data with the negative part of the k-space truncated. The k-space data is denoted as *S*(*k*), the r-space data is denoted as *V*(*r*), and the real part of the image data is denoted as *I*(*r*). The original asymmetric k-space data is denoted as *S*_orig_(*k*): *−K*_c_ ≤ *k* ≤ *K*_max_ *−* 1 (*K*_c_ < *K*_max_), the truncated region (−*K*_max_ ≤ *k* < *−K*_c_) is filled with zeros and the *K*_c_ is truncation parameter. Initially, the following 1D k-space window functions for AFI are defined as:

a) Low-pass symmetric window #1:
  (1) $$H_{\text{low}.\text{back}}(k_{r})=\{\begin{array}{cc} \text{exp}[(-\text{ln}2)\cdot {\left(k_{r}/K_{r2}\right)}^{2}] & :0<|k_{r}|\leq K_{\text{c}},\\ 0 & :\mathrm{otherwise} \end{array}$$
  where *k*_r_ is *k* in 1D data, or radial in polar coordinate in k-space in 2D or 3D data, and the *K*_r2_ is decided dependent only on the phase distribution in r-space and independent of *K*_c_ under *Kr*_2_ *≤ K*_c_ /2, because this window is used for background phase estimation, and thus that is different from the other windows, defined below. This window becomes circular symmetric shape for 2D or 3D data. The *Kr*_2_ and the minimum *K*_c_ must be decided based on the phase distributions for the target sequence and subject. Appropriate setting of this window is one of the keys for ensuring the effectiveness of RepAFI.
b) Low-pass symmetric window #2:
  (2) $$H_{\text{low}}(k)=\{\begin{array}{cc} 1 & :|k|\leq K_{\text{c}}-K_{1},\\ \text{exp}[(-\text{ln}2)\cdot {\left[\{k-2(K_{\text{c}}-K_{1})\}/K_{1}\right]}^{2}] & :K_{\text{c}}-K_{1}<|k|\leq K_{\text{c}},\\ 0 & :\mathrm{otherwise} \end{array}$$
  where *K*_1_ (0<*K*_1_≤*Kc*) is a parameter that determines the range of the flat-top for gain = 1 for |*k*| ≤ *K*_c_ *− K*_1_. This window is used for phase correction in the Margosian technique and other conventional AFI algorithms, but in the RepAFI technique, it is only used for creating the windows as described in c) and d) below.
c) Homodyne high-pass window:
  (3) $$S(k<-K_{\text{c}})=0:H_{\text{high}.\text{homo}}(k)=\{\begin{array}{ll} H_{\text{low}}(k) & :k<0\\ 2-H_{\text{low}}(k) & :k\geq 0 \end{array}$$
  This window enhances the opposite side of the truncated high-frequency k-space region (*k* > *K*_c_) by a factor of 2 and is used to compensate for the truncated k-space region in the Margosian and homodyne techniques.
d) Asymmetric window:
  (4) $$S(k<-K_{\text{c}})=0:H_{\text{whole}}(k)=\{\begin{array}{ll} H_{\text{low}}(k) & :-K_{\text{max}}\leq k\leq 0\\ 1 & :\mathrm{otherwise} \end{array}$$

This window is used to reduce truncation (ringing) artifacts for whole asymmetric data.

Next, we define the Fourier transform (*FT*[ ]), inverse Fourier transform (*IFT* [ ]), and real part (*Re*[ ]) operators. *FT* [ ] transforms the k-space in the range of *−K*_max_ ≤ *k* ≤ *K*_max_ *−* 1 to the r-space in the range of *−R*_max_ ≤ *r* ≤ *R*_max_ *−* 1; *IFT* [ ] is the inverse transformation of *FT* [ ], and *Re*[ ] extracts the real part of its argument. The RepAFI can be implemented in various ways as similar as the several standard AFI algorithms except for the following part of “low-pass windowing.”

**Fig 2.**
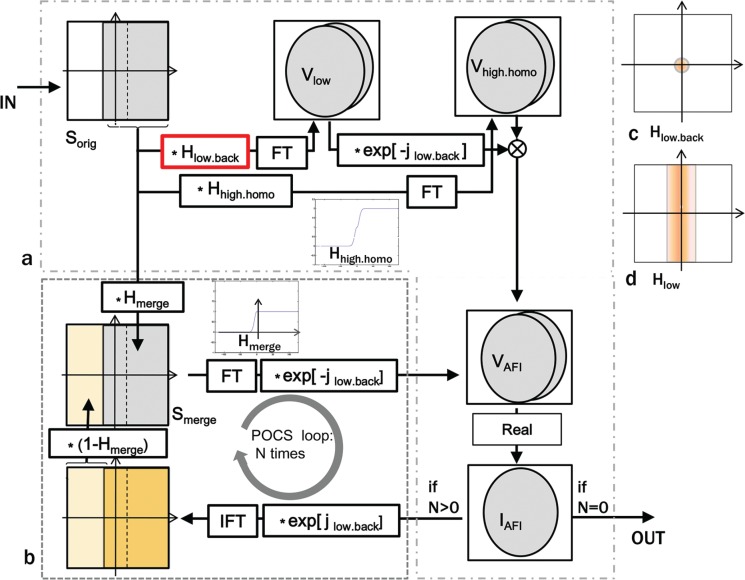
Schematic of the reconstruction process in the real-based polarity-preserving asymmetric Fourier imaging (RepAFI) algorithm. The algorithm consists of Margosian (Homodyne)-based RepAFI without projection on to convex sets (POCS) (*N* = 0) (**a**) and with POCS (*N* > 0) (**b**). Here, *S*_orig_, complex k-space data; *H*_whole_(k), whole asymmetric window; *H*_high.homo_, enhancing high-frequency asymmetric window; *H*_low.back_, low-pass symmetric window; *H*_merge_, merge filter for POCS; FT, Fourier transform; IFT, inverse Fourier transform; and ***Φ***_low.back_, arg[*V*_low.back_]. The shape of the low-pass window in the RepAFI algorithm is the key for correct estimation of the background phase. In standard AFI algorithms, *H*_low_ (**d**) is used instead of *H*_low.back_ (**c**). The details can be referred from the theory section. This flowchart is a modified version of a similar flowchart in reference.^13^

**A. Margosian (Homodyne)-based RepAFI (Fig. 2a)**
  a1) Low-pass windowing:
    $$S_{\text{low}}(k)=H_{\text{low}.\text{back}}(k)\cdot S_{\text{orig}}(k)$$
  a2) $\text{FT}:V_{\text{low}}(r)=FT[S_{\text{low}}(k)]$
  a3) Homodyne windowing:
    $$S_{\text{high}.\text{homo}}(k)=H_{\text{high}.\text{homo}}(k)\cdot S_{\text{orig}}(k)$$
  a4) $\text{FT}:V_{\text{high}.\text{homo}}(r)=FT[S_{\text{high}.\text{homo}}(k)]$
  a5) Phase correction:
    $$V_{\text{AFI}}(r)=V_{\text{high}.\text{homo}}(r)\cdot {\bar{V}}_{\text{low}}(r)/|V_{\text{low}}(r)|$$
  a6) Extraction of the real part in the r-space:
    IAFI(r)= Re[VAFI(r)]
**B. RepAFI with POCS Combination (Fig. 2b)**
  The initial data (*n* = 0) produced by the Margosian-based RepAFI technique (A) is defined as *I*_AFI_(*r*, 0) = *I*_AFI_(*r*). *N* iterations (*n* = 1 to *N*) of steps b1) to b5) given below, where the *N* is decided experimentally, are performed.
  b1) Phase restoration:
    $$V_{\text{AFIrest}}(r,n)=V_{\text{AFIrest}}(r,n-1)\cdot V_{\text{low}}(r)/|V_{\text{low}}(r)|$$
  b2) $\text{IFT}:\;\;V_{\text{AFIrest}}(r,n)=IFT[V_{\text{AFIrest}}(r,n)]$
  b3) Merging of the original and estimated k-space data:
    $$S_{\text{merge}}(k,n)=\{1-H_{\text{merge}}(k)\}\cdot S_{\text{AFIrest}}(k,n)+H_{\text{merge}}(k)\cdot S_{\text{orig}}(k)$$
    Here, it is defined that *H*_merge_ = *H*_whole_.
  b4) $\text{FT}:\;V_{\text{merge}}(r,n)=FT\;[S_{\text{merge}}(k,n)]$
  b5) Phase correction:
    $$V_{\text{AFIrest}}(r,n)=V_{\text{merge}}(r,n)\cdot {\bar{V}}_{\text{low}}(r)/|V_{\text{low}}(r)|$$
  b6) Extraction of the real part in the r-space:
    IAFI(r,n)=Re[VAFI(r,n)]

Note that the main difference between the RepAFI technique and the standard Margosian (Homodyne) technique is the shape of the low-pass filter used for phase estimation. Instead of *H*_low_, which is used in the standard Margosian technique in step a1), *H*_low.back_ is used in the RepAFI technique.

In addition, it is not supposed to contain negative signals in a standard AFI algorithm, final images are usually displayed after taken absolute as |*I*_AFI_(*r*)| to suppress negative signals.

## Materials and Methods

### Simulations

**Fig 3.**
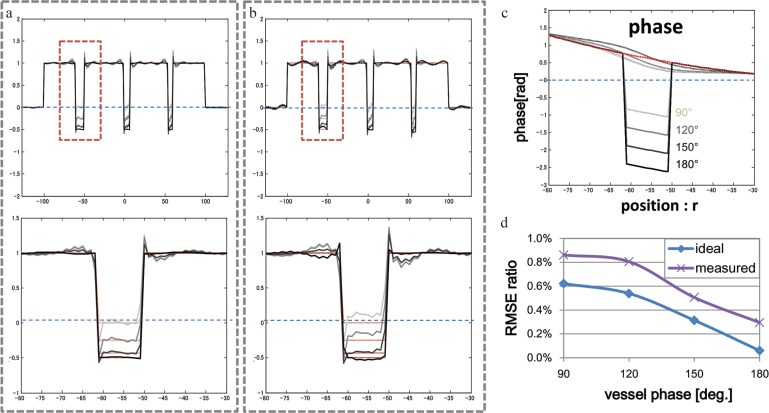
1-dimensional (1D) simulation: dependence on vessel phase. Profiles (whole and magnified, vessel width = 10) corrected with real-based polarity-preserving asymmetric Fourier imaging (RepAFI) using ideal (**a**) and measured (**b**) background phases. The original (solid gray) phase profile (vessel width = 10) and the phase profiles corrected using the ideal (red) and measured (dotted gray) background phases are shown together with various vessel phases (90°, 120°, 150°, and 180° [light to dark]) in (**c**). The root-mean-square-error (RMSE) ratios (**d**) for the profiles increased as the vessel phase difference relative to 180° increased. Note that the errors in the 180° profiles were negligible, but the error was larger for the measured background phase than for the ideal background phase.

Numerical 1D phantom was assumed as shown in Fig. 3, where the background phase of 2nd order *Φ*_back_ = α*r^2^ [rad], −*N*_max_ = < *r* ≤ *N*_max_, (*N*_max_ = 128), and three sizes of blood vessels of width = 10, 8, 5 pixels with the constant phase *Φ*_vessel_ were included in the stationary tissue with rectangular magnitude. The number of data points of full k-space data was 256 (−128 ≤ *k* ≤ 127) and truncated k-space data (*−K*_c_ ≤ *k* ≤ 127) were made after FT of r-space data. The standard parameters were: *Φ*_vessel_ = 180° (ideally inverted); *α* = 0.0002, *K*_max_ = 128, *K*_c_ = 16, *Kr 2* = 4, *K*_1_ = 8, and *K*_2_ = *K*_1_/2. Evaluation items were as follows: a) vessel phase dependency as a parameter of *Φ*_vessel_ = 90°, 120°, 150°, and 180° (ideal); b) *K*_c_ dependency as a parameter of *K*_c_ = 8, 16, 24, and 32; and c) background phase dependency as a parameter of *α* = 0.0001, 0.0002, 0.0003, and 0.0004. In this study, the RepAFI with POCS of *N* = 4 was commonly used.

### MRI experiments

Imaging was performed on 3T whole-body imager (Vantage Titan, Toshiba Medical Systems, Tochigi, Japan) using a 14-ch brain coil for PI. Normal volunteer brain data was obtained according to the regulations of our institution’s internal review board after receiving written informed consent.

First, for comparison among different *K*_c_, two types of 2D IR–FSE with fully sampled data of (**A**) brain axial T_2_-weighted (T_2_W) FLAIR and (**B**) neck axial proton-density weighted (PDW) double IR (DIR) were used. The standard AFI (with POCS) and the RepAFI (with POCS) were also compared for both the data as a parameter of *K*_c_. The low-pass filters for phase estimation in the standard AFI and the RepAFI were respectively “a) Low-pass symmetric window #1” and “b) Low-pass symmetric window #2” as shown in theory section.

**A)** T_2_W-FLAIR 2D-FSE selecting TI < TI_null_ of CSF: TR/TE/TI = 10000 ms/120 ms/2200 ms, ETL=13, 24 slices, slice thickness = 5 mm, FOV = 24 cm, acquisition matrix = 224 (phase encode: x) × 272 (readout: y) (voxel size = 1.1 mm × 0.88 mm), display matrix = 320 × 320 after sinc interpolation, and PI of *R* = 2. For AFI parameters, *K*_c_ was varied and k-space data was truncated the front of phase-encode direction (anterior–posterior for **A** right-to-left for **B**), *Kr*_2_ = 4, *K*_1_ = 8, and *K*_2_ = 4, POCS (*N* = 4) were commonly used.
**B)** PDW DIR BB-2D-FSE selecting TI < TI_null_ of blood: TR/TE/TI = 13000 ms/10 ms/40 ms, ETL = 8, slice thickness = 5 mm, FOV = 20 cm, acquisition matrix = 224 (phase-encode: y) × 384 (readout: x) (voxel size = 0.89 mm × 0.52 mm), display matrix = 384 × 384 after sinc interpolation, no cardiac gating, and the other parameters were same as **A**).

Second, three reconstruction methods of 0-filling, magnitude-based standard AFI, and RepAFI were compared among several TIs (300–600 ms). Although the RepAFI is originally a method that self-data is used for the phase correction, it was not succeeded when the inverted regions were relatively spatially greater than the non-inverted region especially for short TI. Therefore, the phase data of TI = 500 ms was used when TI ≤ 400 ms. The following imaging data was used.

**C)** T_1_W IR–3D-FSE with variable flip angle (3D IR-VFA-FSE):
  TR/TE/TI = 1200 ms/16.5 ms/300–1000 ms, ETL = 32, FOV = 25.6 cm, acquisition matrix = 256 × 256 (voxel size = 1 mm × 1 mm), display matrix = 512 × 512 after sinc interpolation, slice thickness = 3 mm, # of slices = 52, NAQ = 1, PI of reduction-factor (R) = 2 and acquisition time = 4:29. The k-space sampled data, S(*k*_e_, *k*_r_, *k*_s_) was truncated to −12 ≤ *k*_e_ ≤ 128 in the front of phase-encode direction (A-P).

### Evaluation

The AFI algorithms of RepAFI with POCS, respectively, between full sampling and partial sampling were compared visually and quantitatively using an root-mean-square-error (RMSE) ratio, defined for 1D data as:

(5) $$\text{RMSE}=\sqrt{\sum_{r=1}^{R}{\left[I_{\text{AFI}}(r)-I_{\text{full}}(r)\right]}^{2}/R}$$

(6) $$\text{RMSE}\;\text{ratio}=\text{RMSE}/\left[\sum_{r=1}^{R}\left[I_{\text{full}}(r)\right]/R\right]$$

where *R* is a r-space data size, *I*_AFI_(*r*) and *I*_full_(*r*) are respectively the signal intensities for AFI and fully sampled images at the position, *r*. For 2D image data, those were similarly obtained for whole pixels in each 2D image data.

## Results

### Simulations

**Fig 4.**
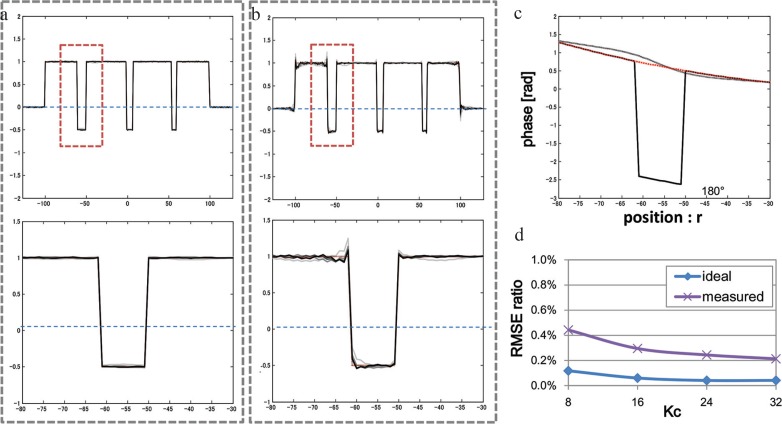
1-dimensional (1D) simulation: dependence on truncation parameter *K*_c_. Profiles (whole and magnified, vessel width = 10) corrected with real-based polarity-preserving asymmetric Fourier imaging (RepAFI) using ideal (**a**) and measured (**b**) background phases for *K*_c_ values of 8, 16, 24, and 32 (light to dark) and the ideal full data (red). The measured background phase (**c**) is independent of *K*_c_ because the low-pass window width used for phase estimation is smaller than the minimum *K*_c_. The root-mean-square-error (RMSE) ratios (**d**) for the profiles decreased as *K*_c_ was increased, but flattened out as *K*_c_ was increased further.

**Fig 5.**
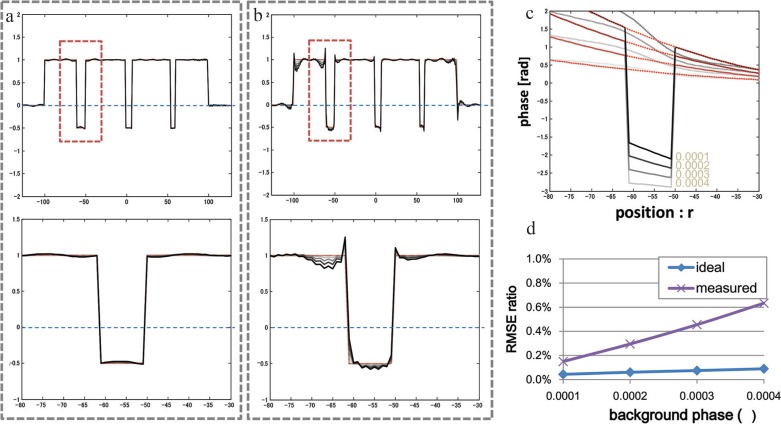
1-dimensional (1D) simulation: dependence on background phase. Profiles (whole and magnified, vessel width = 10) corrected with real-based polarity-preserving asymmetric Fourier imaging (RepAFI) using ideal (**a**) and measured (**b**) background phases for ***α*** values of 0.0001 to 0.0004 (light to dark) shown in (**c**). The root-mean-square-error (RMSE) ratios (**d**) for the profiles increased as ***α*** was increased.

The results for vessel phase dependency, *K*_c_ dependency, and background phase dependency are shown in Figs. 3, 4, and 5, respectively. Errors (RMSE) in the RepAFI profiles were negligible at vessel phase = 180° but were increased with increasing the vessel phase difference from 180° (Fig. 3). Errors in resultant profiles even at vessel phase = 180° were negligible for the ideal phase but not negligible for the phase estimation with low-pass filter particularly around the vessel portions where the phase changes rapidly; and those were decreased with increasing *K*_c_ (Fig. 4) or with decreasing background phase distribution range (Fig. 5).

### MR imaging experiments

**Fig 6.**
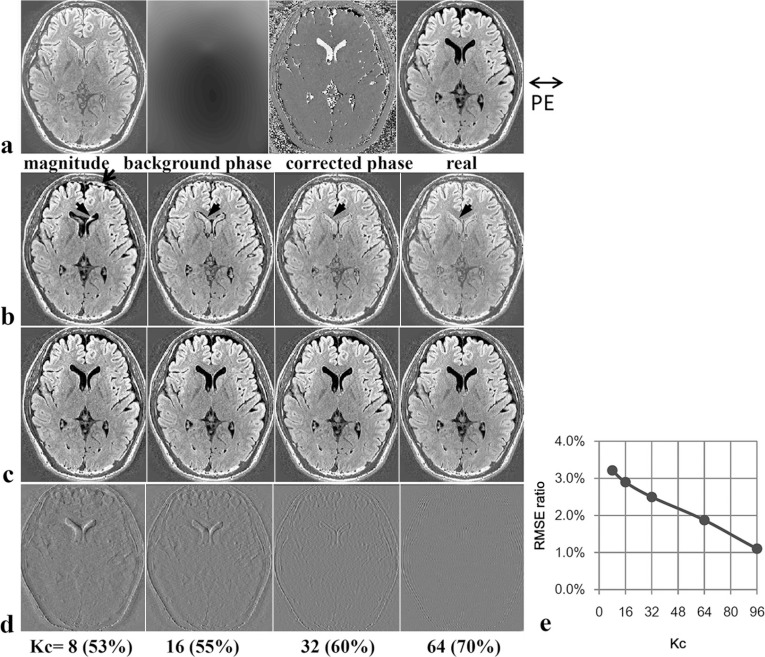
Results for T_2_-weighted (T_2_W)- fluid-attenuated inversion-recovery (FLAIR) brain data. (**a**) Fully sampled (−160 ≤ *k*_y_ ≤ 160) images (from left to right): magnitude image, estimated background phase image (with optimal low-pass filter for real-based polarity-preserving asymmetric Fourier imaging (RepAFI), distributed within *π* [rad] in the whole slice), corrected phase image, and corrected real image. (**b**) Standard AFI images obtained using partial data for several *K*_c_ values. (**c**) RepAFI images obtained using partial data for several *K*_c_ values. (**d**) Images obtained by subtracting the fully sampled corrected real image in the rightmost column of a from the corresponding RepAFI image in (**c**). (**e**) Root-mean-square-error (RMSE) ratio vs. *K*_c_ for (**d**). The errors (indicated by arrows) in standard AFI were corrected by RepAFI. In comparison with the fully sampled real image (top right), *K*_c_ ≥ 8 was necessary to preserve the basic structure in brain tissue, but *K*_c_ ≥ 32 was better for preserving detailed contrast.

**Fig 7.**
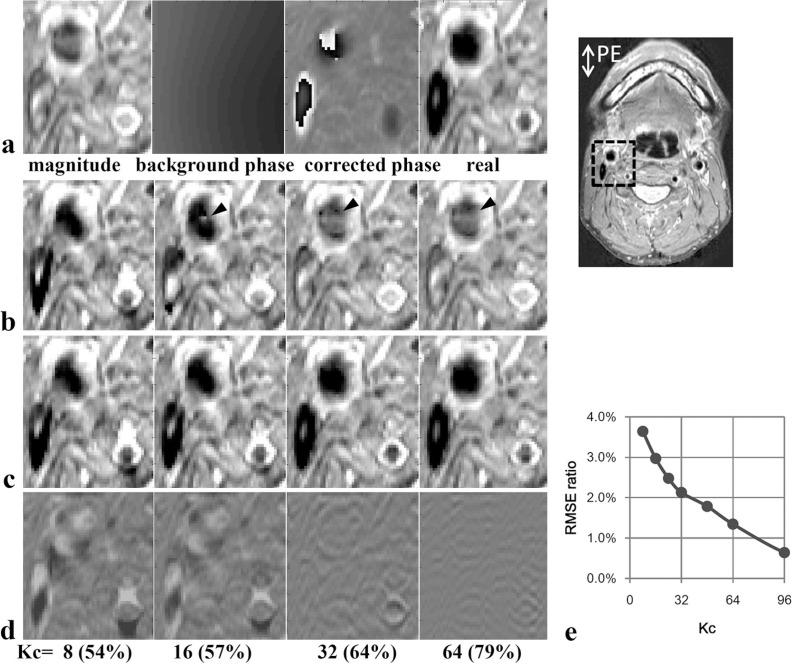
Results for proton-density weighted (PDW) double inversion-recovery (DIR)-fast-spin-echo (FSE) neck data. (**a**) Fully sampled (−112 ≤ *k*_y_ ≤ 112) images (from left to right): magnitude image, estimated background phase image (with optimal low-pass filter for real-based polarity-preserving asymmetric Fourier imaging (RepAFI), distributed in the range of −2.5 to −1.2 [rad] in the region), corrected phase image, and corrected real image. (**b**) Standard AFI images obtained using partial data for several *K*_c_ values. (**c**) RepAFI images obtained using partial data for several *K*_c_ values. (**d**) Images obtained by subtracting the fully sampled corrected real image in the rightmost column of a from the corresponding RepAFI image in (**c**). (**e**) Root-mean-square-error (RMSE) ratio vs. *K*_c_ for (**d**). The errors (indicated by arrows) in the standard AFI were corrected by RepAFI. In comparison with the fully sampled real image (top right), *K*_c_ ≥ 32 was necessary for preserving the vessel shape.

The results of dependency on *K*_c_ for A) T_2_W-FLAIR and for B) DIR–BB–FSE are shown in Figs. 6 and 7, respectively. The standard AFI did not provide correct magnetization polarity with increasing *K*_c_ due to the effects that positive signal regions became wider in CSF regions (Fig. 6) or blood vessel regions (Fig. 7). In contrast, the RepAFI provided similar images as the real images obtained from the fully sampled data, almost independent of *K*_c_ when *K*_c_ was selected greater than the twice of 2D low-pass window size (2*K_r2_ = 8 for seq-A, and = 32 for seq-B) for background phase estimation. For seq-A, it was regarded as the *M*_z_ in CSF sections were almost perfectly inverted to 180° and the background phase was almost correctly estimated in this data; however, for seq-B, the vessel phase might not be perfectly 180°. This could be due to the motion (flow)-induced phase after inversion, because the blood vessel phase becomes |*Φ*_vessel_| < 180° even in the fully sampled data in spite of the background phase being estimated relatively well by the 2D low-pass filter.

**Fig 8.**
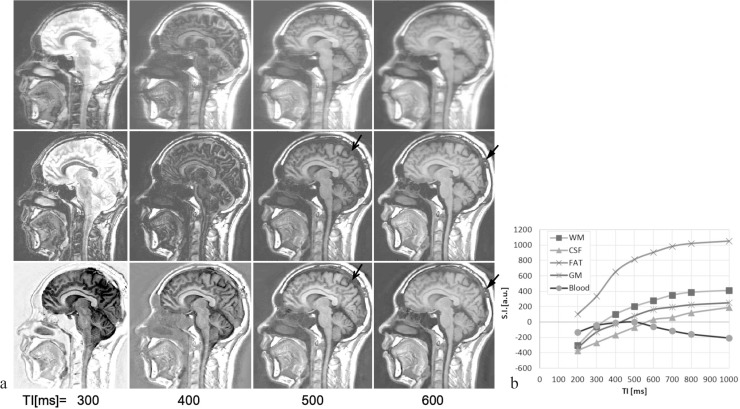
TI dependence of T_1_W 3D IR-VFA-FSE brain images. (**a**) T_1_W 3D IR-VFA-FSE brain sagittal images reconstructed with TI as a parameter. Top row: 0-filling. Middle row: magnitude-based standard AFI. Bottom row: RepAFI. (**b**) Signal intensities as a function of TI for different tissues in the same RepAFI image. Note that the phase of each self-data was used to correct the data for TI ≥ 500 ms, but the phase of TI = 500 ms was used to correct for the data for TI ≤ 400 ms due to the limitation of self-data based phase estimation.

Regarding the results for brain 3D IR–VFA–FSE imaging with TI as a parameter (Fig. 8a: images, b: graph), it can be seen that the blurring in the 0-filling images was corrected in the RepAFI images. Also, the *M*_z_ polarity wraparound in the standard AFI images was corrected in the RepAFI images. In addition, the RepAFI provided correct T_1_W contrasts while preserving the signal polarity. The relationships among the signal intensities of different tissues were not preserved with the standard AFI, but they were preserved for all TI values with RepAFI, irrespective of whether the signal intensities were positive or negative (TI = 300 ms: CSF < GM < WM < 0, TI = 400 ms: CSF < GM < 0 < WM, TI = 500 ms: CSF < 0 < GM < WM, and TI = 600 ms: 0 < CSF < GM < WM).

## Discussion

We proposed a modified AFI technique named RepAFI, in which the background phase is estimated using an optimal low-pass window in the standard AFI algorithm, and assessed thorough simulations and volunteer MR study. It was confirmed that our RepAFI provided *M*_z_-polarity-preserved data nearly equivalent to fully sampled data even when asymmetrically sampled k-space data is used, and in addition, the main requirements for RepAFI are that the background phase estimation should be correct and the inverted portions should be close to 180° as far as possible. Next, we discussed about the limitations and the solutions on RepAFI technique.

### TI dependency in phase estimation

We found that, for actual MR imaging data acquired using IR–FSE sequences, the TI range for successful phase correction was limited to the range where the tissue signals were positive as shown in Fig. 8. This limitation for RepAFI applies when the background phase map is estimated from the self-data with low-pass filter. The limitations related to the TI range and the spatial extent of the negative signal sections in the current low-pass filter-based method used for IR self-data in RepAFI will be reduced by utilizing a better phase estimation technique, such as measurement of the background phase after elimination of the blood vessel and CSF regions.

### Sequence type for RepAFI

It is known that VFA-FSE sequence enables to well suppress the blood vessel signals due to the dephasing effects of variable FA pulses^8^ but it is not sufficient alone. To further enhance BB effects on VFA–FSE, a combination with motion-sensitizing driven equilibrium (MSDE) prepared technique was proposed^9^; however, its minimum signal is zero and sometimes introduces motion artifacts or SNR reduction. Wang’s gradient echo-based PSIR method^7^ requires another data for phase correction. In contrast, the RepAFI with IR–VFA–FSE could enhance vessel-to-background contrasts by keeping blood signal negative by itself if the global signals are positive, without introducing artifacts or SNR reduction.

When our RepAFI is applied to highly asymmetric data (data with smaller *K*_c_) obtained under unignorable motion-induced phase errors such as in neck arteries, as shown in Fig. 7, the use of a shorter TE or a flow-refocusing sequence will further improve the results, because the vessel phase is expected to become closer to 180°.

### Data spatial dimension

Regarding the spatial dimension for RepAFI, the higher the better (3D than 2D) results will be obtained. Our simulation was performed using 1D data and MR imaging were performed using 2D data. The higher dimensional data that RepAFI is actually applied will provide further better estimation for background phase even using the self-data with low-pass filter, since the multi-dimensional low-pass filter enable to effectively suppress the negative-polarity regions such as CSF, which become relatively smaller in higher dimensional image, i.e., 3D will be better than 2D. For 2D partial sampling in 2D data,^11^ low-pass filter shape becomes similar to *H*_low.back_, however, it depends on *K*_c_.

### Background phase estimation using extra data

If it is not easy to correctly and robustly estimate the background phase using the only self-data obtained by IR sequence regardless for full or partial sampling, it is alternative to use separately acquired data of including only background phase such as no IR sequence instead of using self-data; though extra acquisition time is required. In addition, it is desirable to use the same readout conditions including motion correction might become necessary between the main data and the extra data for phase estimation. This will decrease the dependence on the TI and the readout sequence type (SE or GRE), because the background phase can be eliminated without using the spatial frequency differences in the self-data. In addition, even if the high-frequency components in the background phase are significant, such as in the case of GRE sequences, the background phase and negative signal components can be separated out independently, thus enabling perfect correction.

## Conclusion

Although the main limitations are that our proposed RepAFI technique is only applicable to spin-echo-based PSIR sequences and the negative signal structures must be confined to relatively small parts in imaging volume, it achieves a practical balance of image quality and simplicity to provide better performance than conventional AFI methods. It is expected to be especially useful in 3D black-blood vessel wall imaging, although further optimization of parameters for pulse-sequence or clinical evaluation are required.
